# Prognostic Factors for Adrenocortical Carcinoma Outcomes

**DOI:** 10.3389/fendo.2016.00099

**Published:** 2016-07-25

**Authors:** Claudia Scollo, Marco Russo, Maria Antonietta Trovato, Daniela Sambataro, Dario Giuffrida, Mario Manusia, Giulia Sapuppo, Pasqualino Malandrino, Riccardo Vigneri, Gabriella Pellegriti

**Affiliations:** ^1^Endocrinology, Department of Clinical and Experimental Medicine, University of Catania, Catania, Italy; ^2^Endocrinology, Department of Clinical and Experimental Medicine, University of Messina, Messina, Italy; ^3^Surgical Oncology, Garibaldi-Nesima Hospital, Catania, Italy; ^4^Medical Oncology, Garibaldi-Nesima Hospital, Catania, Italy; ^5^Istituto Oncologico del Mediterraneo, Viagrande, Catania, Italy; ^6^Pathology, Garibaldi-Nesima Hospital, Catania, Italy; ^7^Humanitas, Catania Oncology Center, Catania, Italy; ^8^Institute of Biostructures and Bioimaging, CNR, Catania, Italy

**Keywords:** adrenocortical carcinoma, adrenal cancer, prognostic factors, recurrence, survival

## Abstract

**Purpose:**

Adrenocortical carcinoma (ACC) is an aggressive tumor characterized by a high recurrence rate and poor response to treatment. This study analyzes a consecutive series of ACC patients to evaluate the prognostic value of various clinical and pathological characteristics.

**Methods:**

We retrospectively evaluated 32 ACC patients followed at our Medical Center from 1997 to 2015 and evaluated the prognostic value of age at diagnosis, gender, tumor functional status, stage, and type of treatment with respect to overall survival (OS) and disease-free survival (DFS), as determined by Kaplan–Meier curves.

**Results:**

ACC was associated with hormonal overproduction in 50% of cases, and patients with isolated hyperandrogenism had a better prognosis. Recurrence was observed in 12/26 (46.2%) patients with no evidence of disease after surgery. Tumor size [hazard ratio (HR) 1.32, 95% confidential intervals (CI) 1.12–1.64; *p* = 0.007], ki-67 (HR 1.06, 95% CI 1.02–1.11; *p* = 0.009) and advanced stage at diagnosis (III–IV) (HR 6.51, 95% CI 1.65–24.68; *p* = 0.006) were associated with recurrence in the 26 R0 patients in the univariate analysis. Advanced stage was an independent risk factor for recurrence in the multivariate analysis (HR 8.10, 95% CI 1.55–41.35; *p* = 0.01). Five-year survival was 40.0%. Positive resection margins (HR 10.61, 95% CI 3.02–38.31; *p* = < 0.001), ki-67 (HR 1.04, 95% CI 1.01–1.07; *p* = 0.01) and advanced stage (HR 11.31, 95% CI 1.45–87.76; *p* = 0.02) were associated with poor survival in all 32 patients, but only positive resection margins were an independent predictor of mortality in the multivariate analysis (HR 6.22, 95% CI 1.44–26.05; *p* = 0.01).

**Conclusion:**

ACC has a poor prognosis with a high recurrence rate. Tumor stage at diagnosis and the completeness of surgical excision are the most relevant prognostic factors.

## Introduction

Adrenocortical carcinoma (ACC) is a rare tumor with an incidence of approximately 0.7–2 cases/million inhabitants per year (1–3). The clinical presentation is characterized by functional symptoms related to adrenal hormone overproduction (Cushing’s syndrome, hyperandrogenism, and hyperaldosteronism) and/or by local symptoms due to compression by the tumor mass. Sometimes, the diagnosis can be an incidental finding after abdominal imaging.

ACC is one of the most aggressive solid tumors, and complete surgical resection (which implies localized disease) is the curative treatment that offers the best hope for long-term survival. However, ACC remains a challenging malignancy because of the high frequency of recurrent disease and the low rate of response to postsurgical therapies (3–6). The estimated 5-year overall survival (OS) rate ranges from 16 to 44%, decreasing to less than 13% in patients with metastatic disease (3–6).

In spite of promising advances in molecular characterization and therapeutic approaches, the ACC outcome remains poor. Therefore, reporting the clinical experience of even a limited number of cases might be useful in attempts to provide an accurate prognosis and better care for this rare but aggressive disease.

In this retrospective study, we analyzed the clinical and pathological features of a consecutive series of 32 patients affected by ACC followed at our Medical Center. These features were evaluated with respect to their relevance to outcome and prognosis.

## Materials and Methods

### Patient Records and Follow-up

We retrospectively analyzed the clinical characteristics at presentation, treatment, and outcome of a continuous series of 32 ACC patients followed at our Medical Center from 1997 to 2015. Clinical records were reviewed and data on age at diagnosis, gender, functional tumor status (assessed by cortisol, aldosterone, and androgen levels), tumor size, stage at diagnosis as well as treatment details and outcome were analyzed. Some preoperative findings, as radiological reports, were not included in the analysis because they were performed by different centers and with non-standardized procedures.

All patients underwent surgical resection of the ACC *via* the open or laparoscopic approach (on the basis of tumor size or preoperative imaging findings) at the Surgical Oncology Unit of our Medical Center.

Tumor specimens were reviewed by our pathologists and staged according to the ENSAT staging system (7), which defines stage I ACC as measuring ≤5 cm and confined to the adrenal gland, stage II as intra-adrenal ACC >5 cm, stage III by the presence of regional nodal involvement or local invasion (infiltration of surrounding tissue or vascular tumor extension), and stage IV by evidence of distant metastases. Resection margins status (the distance between the tumor and the edge of the surrounding tissue removed with the tumor) was evaluated by reviewing pathological specimens and defined as follows: R0, no evidence of tumor; R1, microscopically positive resection margins; R2, macroscopic residual disease. Ki-67 was also measured in 19 patients.

All patients were followed up at our Medical Center. The postoperative evaluation included adrenal hormonal assessment (urinary free cortisol, androgens, and aldosterone were measured by commercially available competitive enzyme immunoassay or radioimmunoassay with intra- and inter-assay coefficients of variation for all assays <5 and <10%, respectively) and morphological staging (CT or RMN, bone scan, and/or FDG PET scan); in the absence of biochemical and/or radiological signs of persistent/recurrent disease patients were considered free of disease. Adjuvant therapy with mitotane (*o,p*-DDD, Lysodren 500 mg tablets, Laboratoire HRA Pharma, France) was started in 17 patients according to tumor stage and histopathological features of aggressiveness (tumor size >8 cm, ki-67 >10%). This treatment was not given to five patients free of disease but diagnosed before 2001, when mitotane was not available at our Center (3, 8). Adjuvant radiotherapy after surgery was not administered in our patients.

Patients were followed every 3–6 months by clinical, hormonal (in secreting tumors), and imaging evaluations. All patients with persistent/recurrent disease had undergone surgery or systemic therapy (mitotane and/or chemotherapy). Patients treated with mitotane underwent adrenal replacement therapy with cortisone acetate (cortone acetato 25 mg tablets, Teofarma, Italy) and fludrocortisone acetate (florinef 0.1 mg tablets, Bristol–Myers Squibb, Switzerland). Mitotane treatment was started at a low dosage (9) and then increased with the objective of achieving an adequate therapeutic range (14–20 mg/l) (10). Mitotane serum levels were routinely measured using the Lysosafe service provided by HPA Pharma.^1^ In 15 cases presented at the Medical Oncology Unit of our Medical Center, systemic chemotherapy [etoposide/doxorubicin/cisplatin (EDP) regimen] was prescribed for progressive/non-responsive disease. Progressive or stable disease was defined according to RECIST Criteria 1.1. OS time was calculated from the date of diagnosis to the date of death or to the last follow-up for censored patients. Disease-free survival (DFS) was measured in R0 patients from the date of diagnosis to the date of recurrence.

### Statistical Analysis

Quantitative data are shown as mean ± SD, and numbers and percentages are provided for qualitative data. Percentages were compared using chi-squared tests, and Student’s *t*-test was used for continuous variables. Univariate and multivariate analyses were performed using the Cox proportional hazards regression model. The results are reported with hazard ratio (HR) and 95% confidential intervals (CI). The OS and DFS curves were determined by the corresponding Kaplan–Meier curves. All tests were two-sided, and *p* values <0.05 were considered statistically significant. Statistical analysis was performed using SPSS software, version 13.0 for Windows (SPSS Inc., Chicago, IL, USA).

## Results

### Clinical and Hormonal Status at Presentation

Table 1 summarizes the clinical and histopathological features of the 32 consecutive ACC patients (F = 22, M = 10, F/M ratio = 2.2) included in this retrospective study. The mean age at diagnosis was 48.5 ± 16.5 years (median 51.4, range 19.9–78.9 years): younger in females (43.6 ± 15.5) than in males (59.8 ± 14.0; *p* = 0.01) and in patients with secreting tumors (*n* = 16) relative to patients with non-functioning tumors (*n* = 16) (40.2 ± 14.7 vs. 56.7 ± 14.2, respectively; *p* = 0.003).

**Table 1:** **Clinicopathological characteristics in 32 ACC patients**.

	Patients (%)
*N*	32
Male gender	10 (31.3)
Mean age at diagnosis (years) ± SD	48.5 ± 16.5
Mean tumor size (cm) ± SD	9.5 ± 4.7
Functioning tumors	16 (50.0)
Androgens and cortisol	6 (37.5)
Androgens alone	7 (43.7)
Cortisol alone	2 (12.5)
Aldosterone	1 (6.3)
ki-67 >10%	10 (52.6)^a^
R0	26 (81.3)
ENSAT stage	
I	3 (9.3)
II	15 (46.9)
III	7 (21.9)
IV	7 (21.9)

*^a^Ki-67 analysis was performed in 19/32 cases*.

Among the patients with hormone-secreting tumors, most were females (15/22 or 68.2%), with only one case (1/10 or 10.0%) of functioning AAC among the male patients (*p* = 0.006). In particular, androgen secretion was found in seven patients (43.7%). The secretion of cortisol alone was observed in only two patients (12.5%); the secretion of both androgens and cortisol was observed in six patients (37.5%); one case (6.3%) had hyperaldosteronism at diagnosis.

The clinical phenotype caused by the ACC was overt Cushing’s syndrome in five cases (15.2%) and hirsutism and oligoamenorrhea in eight cases (25%). Resistant hypertension with pronounced hypokalemia was found in the patient with the aldosterone-secreting carcinoma. Two patients with secreting tumors were clinically asymptomatic. The non-functioning ACCs were revealed by abdominal discomfort and back pain in 14 (43.7%) patients. Two non-functioning ACCs were diagnosed incidentally during abdominal imaging exams performed for other indications.

In the seven female patients with isolated hyperandrogenism, age at diagnosis was lower in patients with hypercortisolism (alone or in association with androgen issues) and those with non-functioning ACCs (Table 2). In this series, no patient had clinical or laboratory evidence of hereditary cancer syndromes associated with ACC (Li–Fraumeni syndrome, MEN-1, Beckwith–Wiedemann syndrome or familial adenomatous polyposis), although bilateral adrenal ACC was found in one patient.

**Table 2:** **Comparison between the ACC patients subdivided according to the type of endocrine function**.^a^

	Only androgens	Cortisol with or without androgens	Non-functioning
*N*	7	8	16
Age at diagnosis	31.6 ± 7.3	45.7 ± 16.4*	56.7 ± 14.2***
Tumor size	8.9 ± 4.5	10.3 ± 4.8	9.9 ± 5.0
R1–2	0	2	3
Stage III–IV	2	4	7
Persistent/recurrent disease	2	6	9
Progression/death for disease	0	6**	9****

*p = 0.058 vs. androgens secreting tumors.

**p < 0.01 vs. androgens secreting tumors.

***p < 0.001 vs. androgens secreting tumors.

****p < 0.05 vs. androgens secreting tumors.

*^a^One patient secreting aldosterone is not included in this table*.

### Surgical Treatment and Histopathologic Characteristics

Open surgery was performed on the primary tumor in 25 patients (78.1%). In the remaining seven patients (21.9%) with a preoperative diagnosis suspicious of adenoma and an adrenal mass that was small in size (<4 cm), a laparoscopic approach was used for surgery. Negative resection margins (R0) were achieved in 26 patients (81.3%). Positive margins (R1) were found in two patients (6.3%). Macroscopically residual disease (R2) was present in the four remaining patients (12.5%).

Mean tumor size was 9.5 ± 4.7 cm (median 9.0, range 3.5–25.0 cm) with no significant difference according to gender (cm 9.7 ± 5.2 in females vs. 9.4 ± 3.7 in males; *p* = 0.9) or ACC endocrine function (secreting vs. non-secreting tumors, 9.3 ± 4.6 vs. 9.9 ± 5.0, respectively; *p* = 0.7).

Ki-67 values were quite variable, ranging from 2 to 80%; in 10/19 cases (52.6%), the ki-67 value was >10%.

According to the ENSAT staging classification, only 3 patients were classified as stage I, 15 as stage II, 7 as stage III, and 7 as stage IV (Table 1). Distant metastases at diagnosis (M1) in stage IV patients were localized to liver (five patients), lung (six patients), bone (three patients), and peritoneum (one patient). Metastatic disease occurred during follow-up in 18 patients: liver and/or lung were affected in most cases (62.5%), followed by lymph nodes, bone, and peritoneum.

**Figure 1:**
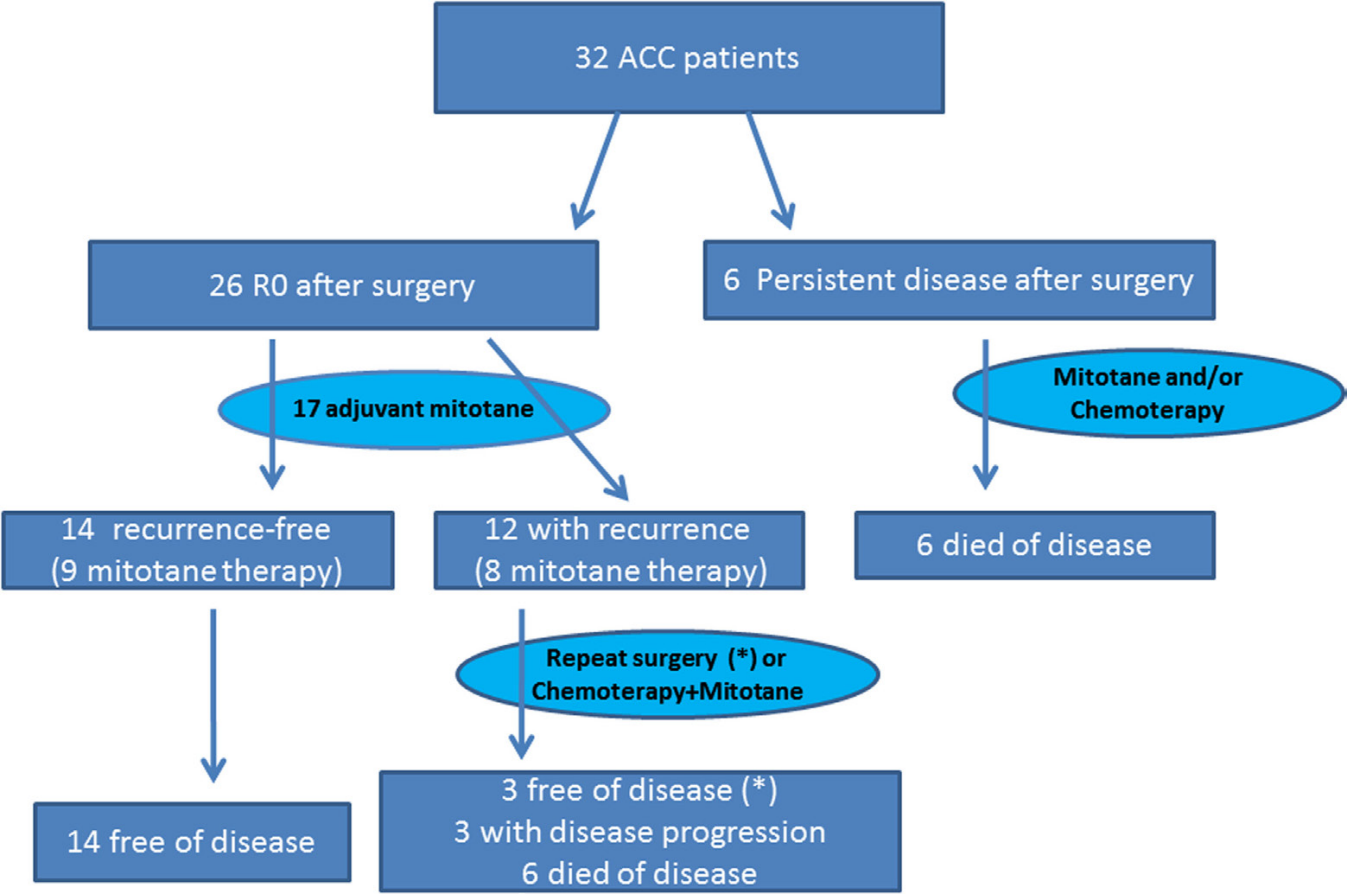
**Treatment and outcome algorithm in our series of 32 ACC patients**. *The three disease-free patients at last control were treated with repeated surgery after recurrence.

After surgery, 26 patients were R0: all patients with stage I, II, and III ACC and one patient with stage IV. Among the remaining six patients (18.8%, all with stage IV disease), tumor debulking but not complete removal of the primary tumor was achieved through surgery (Figure 1).

### Postsurgical Therapy

After surgery, most (17/26 or 65.3%) R0 patients received adjuvant mitotane treatment (1 at stage I, 9 at stage II, 6 at stage III, and 1 at stage IV) (Table 3). Mitotane serum levels in the therapeutic range (14–20 mg/l) were obtained in 10 cases (58.8%), while this target was not reached in the 7 remaining patients (41.2%) (5 patients did not tolerate the drug, and 2 patients had low compliance).

**Table 3:** **Comparison between the 26 R0 patient in relationship to the adjuvant mitotane therapy**.

	Adjuvant mitotane	No adjuvant therapy	*p*
*N*	17	9	
Age at diagnosis	45.1 ± 16.0	53.9 ± 19.9	0.2
Tumor size	9.4 ± 3.8	8.4 ± 4.0	0.5
Functioning tumors	10	3	0.4
Stage III–IV	7	1	0.2
Recurrence	8	4	0.9

Chemotherapy was prescribed for the six R1–2 patients with persistent disease after surgery: a mitotane + EDP regimen in four cases, mitotane alone or EDP alone in the remaining cases.

### Outcome

#### Tumor Recurrence

Median DFS was 78.7 months (95% CI 10.1–147.3). Cancer recurrence was observed in 12 of the 26 R0 patients (46.2%) after a median period of 19.4 months (range 3.0–108.6): 3 of these patients underwent a second surgical intervention because of localized disease, while 9 were treated with EDP in association with mitotane as first-line systemic chemotherapy.

ACC recurrence occurred in 8/17 R0 patients (47.1%) under adjuvant mitotane therapy and in 4/9 (44.4%) R0 patients not treated with mitotane (Table 3). The recurrence rate was not different among the 10 mitotane-treated patients that reached the therapeutic range (recurrence in 5/10) as compared to the 7 patients that did not reach that target (recurrence in 3/7).

**Figure 2:**
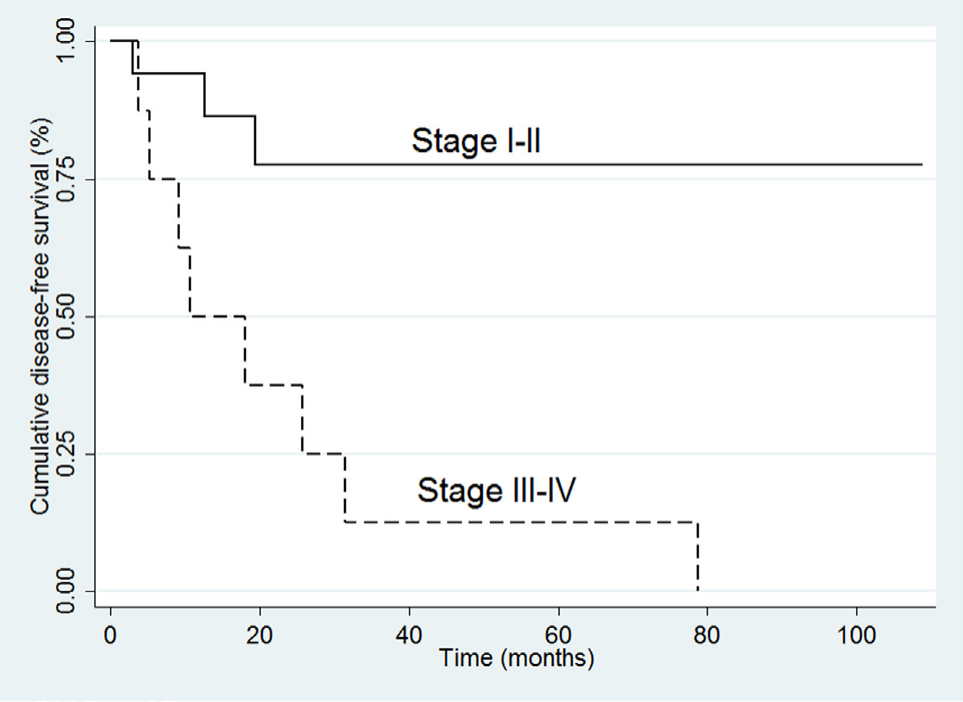
**Kaplan–Meier survival curves for disease-free survival in the 26 R0 patients**.

In the 26 R0 patients, the univariate analysis indicated that ACC recurrence was positively associated with tumor size (HR 1.32, 95% CI 1.12–1.64; *p* = 0.007), ki-67 (HR 1.06, 95% CI 1.02–1.11; *p* = 0.009), and advanced stage at diagnosis (III–IV vs. I–II: HR 6.51, 95% CI 1.65–24.68; *p* = 0.006) (Table 4). The multivariate analysis confirmed that advanced stage at diagnosis was an independent risk factor for recurrence (HR 8.10, 95% CI 1.55–41.35; *p* = 0.01) (Table 4 and Figure 2).

**Table 4:** **Cox proportional model for clinical-pathological parameters associated with recurrence in 26 R0 patients**.

	Univariate analysis		Multivariate analysis^a^	
Parameters	HR (95% CI)	*p*	HR (95% CI)	*p*
Male gender	2.51 (0.69–9.23)	0.2		
Age at diagnosis	0.97 (0.92–1.12)	0.8		
Functioning tumors	0.88 (0.19–2.56)	0.7		
Mitotane therapy	1.42 (0.25–6.54)	0.7		
Tumor size	1.32 (1.12–1.64)	0.007		
Ki-67	1.06 (1.02–1.11)	0.009		
Stage III–IV	6.51 (1.65–24.68)	0.006	8.10 (1.55–41.35)	0.01

*^a^Adjusted for gender, age at diagnosis, functioning tumors, mitotane therapy, and stage. Ki-67 was not included in multivariate analysis since it was not available for all cases*.

#### Patient Survival

At the last control visit, after a median follow-up of 29.3 months (range 4.3–189.9), 17 patients (53.1%) were disease-free (3/3 stage I, 12/15 stage II, 1/7 stage III, and 1/7 stage IV), while 3 patients (2/15 stage II and 1/7 stage III) showed progressive disease. Twelve patients died from the disease (1/15 stage II, 5/7 stage III, and 6/7 stage IV). ACC patients with isolated hyperandrogenism had the lowest rate of progression/death for the disease compared with cortisol-secreting (*p* < 0.01) and non-functioning ACC patients (*p* < 0.05) (Table 2).

Overall survival at 3 and 5 years for the entire cohort was 50.0 and 40.0%, respectively. At 5 years, OS was 85.7% for stage I–II patients, but only 16.7% for patients at stage III and 14% for patients at stage IV.

The median OS for the entire cohort (*n* = 32) was 55.8 months (95% CI 9.0–97.3). For the six patients with persistent disease after surgery, median OS was only 7.7 months (95% CI 1.2–19.9) (Figure 1). Among the 12 R0 patients with recurrent disease, 3 (all with repeated surgery) were disease-free at the last control visit; 3 had progressive disease, and 6 had died. All 14 R0 patients without tumor recurrence during follow-up were disease-free at the last control visit.

**Figure 3:**
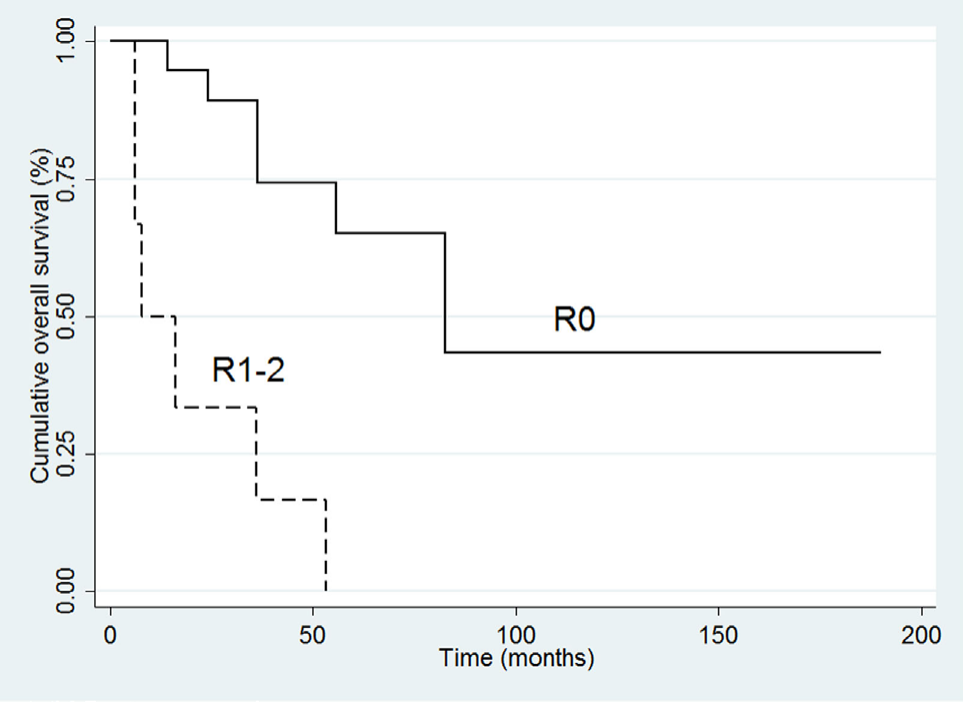
**Kaplan–Meier survival curves for overall survival in all 32 patients**.

In the complete series of 32 patients, the univariate analysis indicated that poor survival was associated with positive margins of resection (HR 10.61, 95% CI 3.02–38.31; *p* < 0.001), ki-67 (HR 1.04 95% CI 1.01–1.07; *p* = 0.01), and advanced stage (III–IV) at diagnosis (HR 11.31, 95% CI 1.45–87.76; *p* = 0.02). At multivariate analysis, only resection margin positivity was an independent predictor of mortality (HR 6.22, 95% CI 1.44–26.05; *p* = 0.01) (Table 5; Figure 3).

**Table 5:** **Cox proportional model for clinicopathological parameters associated with mortality**.

	Univariate analysis		Multivariate analysis^a^	
Parameters	HR (95% CI)	*p*	HR (95% CI)	*p*
Male gender	3.12 (0.88–10.11)	0.06		
Age at diagnosis	1.01 (0.90–1.15)	0.2		
Functioning tumors	0.49 (0.22–1.63)	0.2		
Mitotane therapy	0.65 (0.21–2.60)	0.6		
Tumor size	1.14 (0.93–1.24)	0.1		
≥8 cm	3.09 (0.77–11.49)	0.09		
Ki-67	1.04 (1.01–1.07)	0.01		
R1–2	10.61 (3.02–38.31)	<0.001	6.22 (1.44–26.05)	0.01
Stage III–IV	11.31 (1.45–87.76)	0.02		

*^a^Adjusted for gender, age at diagnosis, functioning tumors, mitotane therapy, R status and stage. Ki-67 was not included in multivariate analysis since it was not available for all cases*.

## Discussion

This study retrospectively analyzes a continuous series of ACC patients, all followed at a single Center from 1997 to 2015. The female/male ratio was 2.2:1, similar to that of other reports (4, 5). The mean age at diagnosis was significantly lower in females. This gender difference could be the consequence of the role of estrogens in adrenal tumorigenesis. Suggested mechanisms include the enhancement of adrenocortical cell proliferation mediated by 17β-estradiol (11), the high expression of estrogen-related receptor α (ERRα) in ACC tissues relative to normal adrenal cortex and to benign adrenal tumors (12), and the relevant role played by ERRα in 17β-estradiol and IGF-II-dependent ACC cell proliferation (13). These lines of evidence make ERRα a promising target for ACC therapy (14).

In our series, the ACC was functioning in 50.0% of patients, more frequently in women (68.2%), as already reported (15). In patients with functional ACC, the age at diagnosis was significantly lower, a likely consequence of the clinical signs and symptoms of hyperandrogenism, such as oligomenorrhoea or hirsutism, which made the diagnosis more evident in women. Since early diagnosis usually corresponds to stage I–II ACC, which has a better prognosis, many studies have found that functional status is an important prognostic factor. In our series, hormonal status was not a relevant prognostic factor, but subdividing patients according to the type of hormonal secretion revealed that patients with isolated hyperandrogenism had better outcomes during follow-up compared with patients with cortisol (alone or in combination with androgen)-secreting ACCs (Table 2). The less favorable prognosis of cortisol-secreting tumors is probably secondary to the immunosuppressive effect of the high cortisol levels (15, 16).

In contrast to previously published studies, in which most patients had ACC in advanced stages, in recent years, a shift toward more precocious stages, with the highest percentage of patients in stage II, has been observed. This is a likely consequence of the increased diffusion of radiological imaging procedures (17). However, despite earlier diagnosis, ACC is associated with a high percentage of recurrences even when surgical resection, the only curative approach, is apparently complete (17, 18).

The ACC recurrence rates reported in the literature are widely variable, ranging from 21 to 91% (19). This variability reflects the heterogeneity of the different series studied. Studies are often affected by selection bias and may include patients referred to specialized centers only after the development of recurrence (17). It is documented, in fact, that the management of ACC patients at highly specialized centers with more than 10 ACC cases treated each year is associated with improved survival and low recurrence rates (20).

ACC remains an aggressive cancer that can recur at any time, although most recurrences occur in the first 2 years after surgery (17). In our series, median recurrence time was 19.4 months, but the disease-free interval after surgery was quite variable (3.0–108.6 months). Tumor size, ki-67 index, and tumor stage are the risk factors significantly associated with recurrence (Table 4). In our series, adjuvant therapy with mitotane did not cause a significant benefit in terms of recurrence, but the small number of patients analyzed the possibility of selection bias, and unidentified interfering factors in the retrospective study do not allow strong conclusions. In a large series, Terzolo et al. have recently observed that adjuvant therapy with mitotane has an independent positive effect on recurrence-free survival (21). Therefore, in patients that are at high risk of recurrence (ki-67 >10%, stage III–IV, tumor size >8 cm), mitotane is strongly recommended (3, 8, 22). The ki-67 proliferation index, assessed by immunohistochemistry, is a validated index of cell proliferation, and it is considered the most predictive histopathologic parameter of recurrence-free survival (3, 8, 22). Also our data (that showed a 6% additional risk of recurrence for each percentage point of ki-67 increase) confirm this relationship. A multicentric European study highlighted that a ki-67 threshold value at 10% represents a cut off to separate patients at low vs. high risk of recurrence (23).

In the high-risk patients, some evidences indicate that adjuvant radiotherapy in the tumor bed after surgery significantly improved local control in resected ACC patients (24).

In low-risk patients, the indications for mitotane therapy are uncertain: current recommendations suggest assigning these patients to the randomized ADIUVO trial^2^ that compares mitotane treatment with a wait-and-see strategy (18, 22). Also, recurrence may occur among low-stage ACC patients: in our series, 4/15 (26.7%) patients with stage II ACC experienced recurrence.

The optimal management of patients with recurrent ACC is not well established. A recent study showed that additional surgical procedures should be performed at first recurrence in patients with a DFS >12 months, when the tumor is completely resectable. In contrast, when recurrence occurs fewer than 6 months after disease diagnosis, the suggested treatment is aggressive medical therapy (25). In our series, three patients with cancer recurrence at 10.6, 19.4, and 31.4 months benefited from repeated surgery and were disease-free at the last control visit.

Despite an increased number of surgical procedures and earlier diagnosis, the survival rate of ACC patients has not significantly improved in recent years: the Netherlands Cancer Registry shows an average overall 5-year survival rate of 32% from 1993 to 2010; this figures jumps to 62% in patients with localized disease (6) and drops to 13% in patients with distant metastases (4, 15). The lack of improvement of OS during recent years is mainly due to the absence of new and effective therapies (26). In our series, the survival rate at 5 years was 40.0%. The individual prognosis largely depended on tumor stage, with OS decreasing markedly from 85.7% for stage I–II to 16.7 and 14% for patients at stages III and IV, respectively. These percentages are similar to those reported in larger series (2–6). However, multivariate analysis showed that incomplete tumor resection was the only independent parameter associated with mortality, highlighting that, at present, radical surgery is the only potentially curative therapy for this disease (27).

Our report and clinical experience remark that ACC patient prognosis is poor and largely depends by tumor stage at diagnosis, with strong relation with postoperative stadiation (e.g. margins status). The rate of recurrence is high. No effective medical therapies are available at present, and the current treatment with mitotane and conventional chemotherapy provides small benefits in most cases.

Recently, genomic studies led to the identification of genetic and epigenetic alterations characterizing subgroups of tumors with activation of specific molecular pathway patterns and with different clinical outcome. Based on these analyses, the prognostic stratification is more accurate than that obtained with the use of traditional prognostic factors. The use of genome-wide expression profile studies, microRNA, and methylation profile may allow a better prognostic prediction and promote the development of molecular-targeted therapies (28, 29).

Our study presents some limitations, the most important being the small series of patients, the retrospective design, and the analysis of prognostic factors restricted to traditional histological markers with no insight on the molecular profile of ACC.

## Conclusion

- ACC is a rare disease, more frequent in women who have a younger mean age at diagnosis and a higher rate of functioning tumors.
- Functioning ACC are diagnosed earlier, but only ACC patients with isolated hyperandrogenism have a better prognosis, not patients with ACC secreting cortisol (alone or in combination with androgens).
- The completeness of surgery, without margin positivity, is a critical requirement to improve outcome. However, the recurrence rate is high, even when surgical resection is apparently complete. Tumor size, ki-67, and a high tumor stage are the risk factors significantly associated with ACC recurrence.

## Author Contributions

MR, CS, GS, PM, RV, and GP treated the patients, gathered data, and drafted the manuscript. MAT treated the patients, carried out the surgeries, and critically reviewed the manuscript. DS and DG treated the patients and critically reviewed the manuscript. MM analyzed histological specimens and critically reviewed the manuscript. All authors contributed to the conception of the work and approved the final version of the manuscript.

## Conflict of Interest Statement

The authors declare that the research was conducted in the absence of any commercial or financial relationships that could be construed as a potential conflict of interest.

## Footnotes

^1^www.lysosafe.com

^2^https://clinicaltrials.gov/ct2/show/NCT00777244
